# Preoperative CT Radiomics Predicting the SSIGN Risk Groups in Patients With Clear Cell Renal Cell Carcinoma: Development and Multicenter Validation

**DOI:** 10.3389/fonc.2020.00909

**Published:** 2020-07-28

**Authors:** Yi Jiang, Wuchao Li, Chencui Huang, Chong Tian, Qi Chen, Xianchun Zeng, Yin Cao, Yi Chen, Yintong Yang, Heng Liu, Yonghua Bo, Chenggong Luo, Yiming Li, Tijiang Zhang, Rongping Wang

**Affiliations:** ^1^Medical College, Guizhou University, Guiyang, China; ^2^Department of Medical Records and Statistics, Guizhou Provincial People's Hospital, Guiyang, China; ^3^Department of Radiology, Guizhou Provincial People's Hospital, Guiyang, China; ^4^Guizhou Provincial Key Laboratory of Intelligent Medical Image Analysis and Precision Diagnosis, Guizhou Provincial People's Hospital, Guiyang, China; ^5^Research Collaboration Department, R&D Center, Beijing Deepwise & League of PHD Technology Co. Ltd, Beijing, China; ^6^Department of Pathology, Guizhou Provincial People's Hospital, Guiyang, China; ^7^Department of Radiology, Affiliated Hospital of Zunyi Medical University, Zunyi, China; ^8^Department of Pathology, Affiliated Hospital of Zunyi Medical University, Zunyi, China; ^9^Department of Urinary Surgery, Guizhou Provincial People's Hospital, Guiyang, China

**Keywords:** clear cell renal cell carcinoma, SSIGN score, prognostic prediction, computed tomography, radiomics

## Abstract

**Objective:** The stage, size, grade, and necrosis (SSIGN) score can facilitate the assessment of tumor aggressiveness and the personal management for patients with clear cell renal cell carcinoma (ccRCC). However, this score is only available after the postoperative pathological evaluation. The aim of this study was to develop and validate a CT radiomic signature for the preoperative prediction of SSIGN risk groups in patients with ccRCC in multicenters.

**Methods:** In total, 330 patients with ccRCC from three centers were classified into the training, external validation 1, and external validation 2 cohorts. Through consistent analysis and the least absolute shrinkage and selection operator, a radiomic signature was developed to predict the SSIGN low-risk group (scores 0–3) and intermediate- to high-risk group (score ≥ 4). An image feature model was developed according to the independent image features, and a fusion model was constructed integrating the radiomic signature and the independent image features. Furthermore, the predictive performance of the above models for the SSIGN risk groups was evaluated with regard to their discrimination, calibration, and clinical usefulness.

**Results:** A radiomic signature consisting of sixteen relevant features from the nephrographic phase CT images achieved a good calibration (all Hosmer–Lemeshow *p* > 0.05) and favorable prediction efficacy in the training cohort [area under the curve (AUC): 0.940, 95% confidence interval (CI): 0.884–0.973] and in the external validation cohorts (AUC: 0.876, 95% CI: 0.811–0.942; AUC: 0.928, 95% CI: 0.844–0.975, respectively). The radiomic signature performed better than the image feature model constructed by intra-tumoral vessels (all *p* < 0.05) and showed similar performance with the fusion model integrating radiomic signature and intra-tumoral vessels (all *p* > 0.05) in terms of the discrimination in all cohorts. Moreover, the decision curve analysis verified the clinical utility of the radiomic signature in both external cohorts.

**Conclusion:** Radiomic signature could be used as a promising non-invasive tool to predict SSIGN risk groups and to facilitate preoperative clinical decision-making for patients with ccRCC.

## Introduction

Renal cell carcinoma (RCC) is the most common malignancy of the kidney in adults, among whom clear cell renal cell carcinoma (ccRCC) accounts for 70–80% of all renal carcinomas (1, 2). This is the most prevalent histological subtype. Surgery is the primary treatment for ccRCC, but about 20–30% of patients will experience metastasis or a recurrence after surgery, and not all of them will benefit from the surgery (3, 4). Therefore, the preoperative risk stratification of patients with ccRCC is increasingly significant from the perspective of personalized medicine. The use of the Stage, Size, Grade, and Necrosis (SSIGN) score is one of the most common prognostic models for ccRCC, and it is a scoring system developed by the Mayo Clinic Center. This is based on the tumor staging, size, grade, and necrosis being used to predict the survival and metastasis rate for ccRCC (5, 6). According to the latest research done by Correa et al. and Shao et al., the SSIGN scoring system shows the best predictive performance in both retrospective and prospective studies relative to other prognostic models (7, 8). However, the clinicopathological data for the SSIGN score is only available after the postoperative pathological evaluation. Therefore, a non-invasive, accurate prediction method of the SSIGN risk group preoperatively may provide great help in the assessment of tumor aggressiveness and the personal management of ccRCC patients.

Computed tomography (CT) is recommended as the first-line assessment tool preoperatively (9). Nevertheless, its efficacy is limited in tumor staging which may lead to an under-staging or over-staging for a considerable proportion of ccRCC patients (4). Radiomics, as an emerging field, refers to transforming medical images into mineable high-throughput feature sets and explores the relationships between these features and the underlying phenotypes to improve clinical decision-making (10). Recently, studies on radiomics have reported that it can be used to predict the RCC from benign renal neoplasms, to classify the subtype of RCC, to discriminate the stages of ccRCC as determined by the World Health Organization/International Society of Urological Pathology (WHO/ISUP), to differentiate sarcomatous transformation, and to predict the Von Hippel–Lindau mutation in ccRCC (11–15). However, previous radiomic studies assessing the invasiveness of ccRCC only focused on the prediction of a single risk index and were limited by unsatisfactory predictive accuracy, small sample sizes, and the absence of multicenter validation. Additionally, to our knowledge, a radiomic signature that can preoperatively predict the SSIGN risk groups in ccRCC has not been reported, to date.

Consequently, the study aims to develop and validate an easy-to-use radiomic signature in multicenter cohorts for a preoperative prediction of the low-risk and the intermediate to high-risk groups based on the SSIGN scores.

## Materials and Methods

### Participant Selection

This was a multicenter retrospective study. All patients with ccRCC were selected from two Chinese hospitals [Guizhou Provincial People's Hospital (GZPPH; Guiyang, China) between August 2013 and December 2017 and the Affiliated Hospital of Zunyi Medical University (AHZMU; Zunyi, China) between February 2010 and December 2017] and the Cancer Genome Atlas (TCGA) database (https://cancergenome.nih.gov), which is currently the largest and most comprehensive public cancer database. Permission for the study was granted by the ethics committee of GZPPH, and the requirement for patient informed consent was waived because it was a retrospective study.

The inclusion criteria were as follows: (1) patients who had confirmed ccRCC by postoperative pathology; (2) patients who did not receive biopsy or any treatment prior to surgery; and (3) pretreatment contrast CT image including at least the nephrographic phase conducted within 30 days before surgery. The exclusion criteria were as follows: (1) patients that received needling biopsy prior to CT examination or any other treatment prior to surgery; (2) no nephrographic phase contrast-enhanced CT images; (3) insufficient CT quality that could not be subjected to analysis (e.g., owing to artifacts or obvious noise); and (4) incomplete demographic or clinicopathology data.

### Demographic and Clinicopathology Data

The age, gender, tumor size, tumor necrosis, T stage, N stage, and TNM stage were obtained from the electronic medical records system and The Cancer Genome Atlas-Kidney Renal Clear Cell Carcinoma (TCGA-KIRC) (16, 17). A new grading system of WHO/ISUP was recommended for ccRCC because Fuhrman nuclear grading was characterized by strong subjectivity and poor repeatability. Therefore, the nuclear grading for all cases was reviewed by two subspecialized genitourinary pathologists (B.Y.H. and Y.Y.T., with 14 and 21 years' experience, respectively) according to the WHO/ISUP grading.

### SSIGN Score Risk Groups

As per the previous clinical study, ccRCC patients were classified into two groups by the SSIGN score according to T stage, tumor size, nuclear grade, and necrosis, as follows: low-risk group (0–3) and intermediate- to high-risk group (≥4) according to the SSIGN score (5).

### Image Feature Analysis

Nephrographic phase contrast-enhanced CT images were downloaded from the image archiving and communication system and The Cancer Imaging Archive (TCIA), https://wiki.cancerimagingarchive.net/) (16). Detailed description of the CT scan equipment and parameters used in the above for both hospitals are shown in Supplementary S1.

The image semantic features were analyzed by two senior radiologists (L.H. and Z.X.C., with 11 and 19 years' experience in imaging diagnosis, who were both kept ignorant of the clinicopathological information except for them being aware of the diagnosis of ccRCC. The image features assessed were as follows: tumor boundary (defined margin or ill-defined margin); necrosis imaging (negative or positive, non-enhanced area is approximately more than 50% of the total tumor); renal vein invasion (negative or positive, tumor thrombogenesis is seen in renal vein or inferior vena cava); collecting system invasion (negative and positive, tumor infiltration of the renal pelvis and renal cone); intra-tumoral vessels (negative or positive, visible vascular enhancement within tumor); lymph node metastasis (negative or positive, peri-renal, hilar, and retroperitoneal lymph nodes >10 mm in the short-axis diameter); visual relative enhancement (hyperattenuating, isoattenuating, and hypoattenuating, compared with the degree of renal cortical enhancement); and enhancement pattern [homogeneity (90%), relative homogeneity (75–90%), and heterogeneity (<75%), in terms of the tumor enhanced homogeneity].

### Tumor Segmentation

The segmentation was executed using the ITK-SNAP version 3.8 software (www.itksnap.org). First, a radiologist (T.C.) with 6 years' experience in abdominal diagnosis was responsible for manually delineating the region of interest (ROI) of the tumor on each slice of the CT nephrographic images by excluding the adjacent vessels, peri-renal fat, and renal parenchyma. Then, these drawn ROIs were reviewed by a senior radiologist (Z.X.C). Any disagreement was determined through mutual negotiation between both radiologists who were kept ignorant of the clinicopathological information.

### Radiomic Feature Extraction

Radiomic feature extraction was accomplished using an open-source python package Pyradiomics with the delineated ROIs (18). To eliminate the impact of the different datasets owing to inhomogeneous CT scanners and parameters, image standardization was implemented as follows: B-spline interpolation resampling techniques were used to standardize the image scale in the slice, resulting in a pixel size of 0.75 mm × 0.75 mm × 0.75 mm. Based on the original images, six common feature groups [(first-order features based on the voxel intensity, shape features, and texture features including the gray-level co-occurrence matrix (GLCM), gray-level run length matrix (GLRLM), gray-level size zone matrix (GLSZM), and gray-level dependence matrix (GLDM)] were extracted. Moreover, the first-order features and texture features were also extracted from two types of filtered images (logarithm and wavelet transformation) from the original CT image. Detailed definitions of the above-extracted texture features can be found in the Pyradiomics documentation.

The feature extraction algorithms were standardized by referring to the Image Biomarker Standardization Initiative (IBSI) (19). In total, 1,218 radiomic features for each region of interest (ROI) of the tumor were extracted from the three-dimensional tumor region. In addition, these extracted features were normalized by the z-score method based on the parameters calculated in the training set in order to standardize the feature values to a normal distribution.

### Inter-observer and Intra-observer Agreement Assessment

The reproducibility of intra-observer and inter-observer agreement for the radiomic features was measured using 45 of patients randomly chosen from three databases. To evaluate intra-observer agreement, the radiomic features extracted from the ROI were delineated by observer 1 (Radiologist T.C.) around 2 weeks using the same method. The inter-observer agreement was assessed by comparing the radiomic features extracted from the ROI as outlined separately by observer 1 first and then by observer 2 (radiologist Z.X.C.). The intra-class correlation coefficient (ICC) was used to evaluate the intra-observer and inter-observer agreement, and the ICC > 0.75 indicated satisfactory agreement and so these were retained for feature selection.

### Radiomic Signature Construction

To minimize overfitting or selection bias in our radiomic features, the least absolute shrinkage and selection operator (LASSO) regression method fit for regression of high-throughput data was utilized to filter the features that best predicted the SSIGN score. The features that remained after LASSO regression were applied to build a radiomic signature by the logistic regression (LR) model through a linear combination of selected features weighted by LR coefficients in the training set. Afterwards, a radiomic score (Rad score) based on the above model formula was calculated for each patient and the cutoff value was statistically analyzed using the Youden index. Finally, the verification of the radiomic signature was performed among the external validation cohorts.

### Image Feature Model and Fusion Model Construction

Univariate and multivariate logistic regressions were in succession used to select the risk factors of the image features for predicting the SSIGN risk group, and the features with *p* < 0.05 were introduced into a multivariate logistic regression to build an image feature model in the training cohort. Additionally, a fusion model was used to integrate the radiomic signature and the independent image features in order to predict the SSIGN score through a multivariate logistic regression model in the training set. In the end, the image feature model and the fusion model were both verified in the external validation cohorts.

### Multicenter Model Validation and Assessment

The predictive value of the radiomic signature, the image feature model, and the fusion model were assessed among the training cohort (*n* = 132), external validation cohort 1 (*n* = 123), and external validation cohort 2 (*n* = 75) regarding discriminability, calibration, and clinical value. The discriminability performance was carried out by the area under the receiver operator characteristic (ROC) curve (AUC), and the differences in AUC values between the three models were compared using the Delong test. The Hosmer–Lemeshow test was used with a calibration curve to determine the goodness of fit. Decision curve analysis (DCA) was used to calculate the net benefits for a range of threshold probabilities in both validation datasets to estimate whether the models was sufficiently robust for clinical use.

### Statistical Analysis

Statistical tests were performed using SPSS (version 21.0, IBM) and R statistical software (version 3.6.0, https://www.r-project.org) or Python (version 3.6.8, https://www.python.org). Univariate analysis was applied to compare the differences of the image feature factors between the two groups by using the chi-square test or Fisher exact test for categorical variables and the Mann–Whitney U-test for continuous variables, where appropriate. The “glmnet” package was used to perform the LASSO regression model analysis. Calibration curve plots were performed using the “gbm” package, and the Hosmer–Lemeshow test was performed using the “generalhoslem” package. Differences in the AUC values between different models were estimated using the DeLong test. The DCA was performed using the “dca.R.” package. The discrimination metrics of the established models, including the AUC, classification accuracy, sensitivity, and specificity were also calculated, and the ROC curves were plotted using Python. A two-sided *p* < 0.05 was considered significant.

## Results

### Patient Characteristics

**Figure 1:**
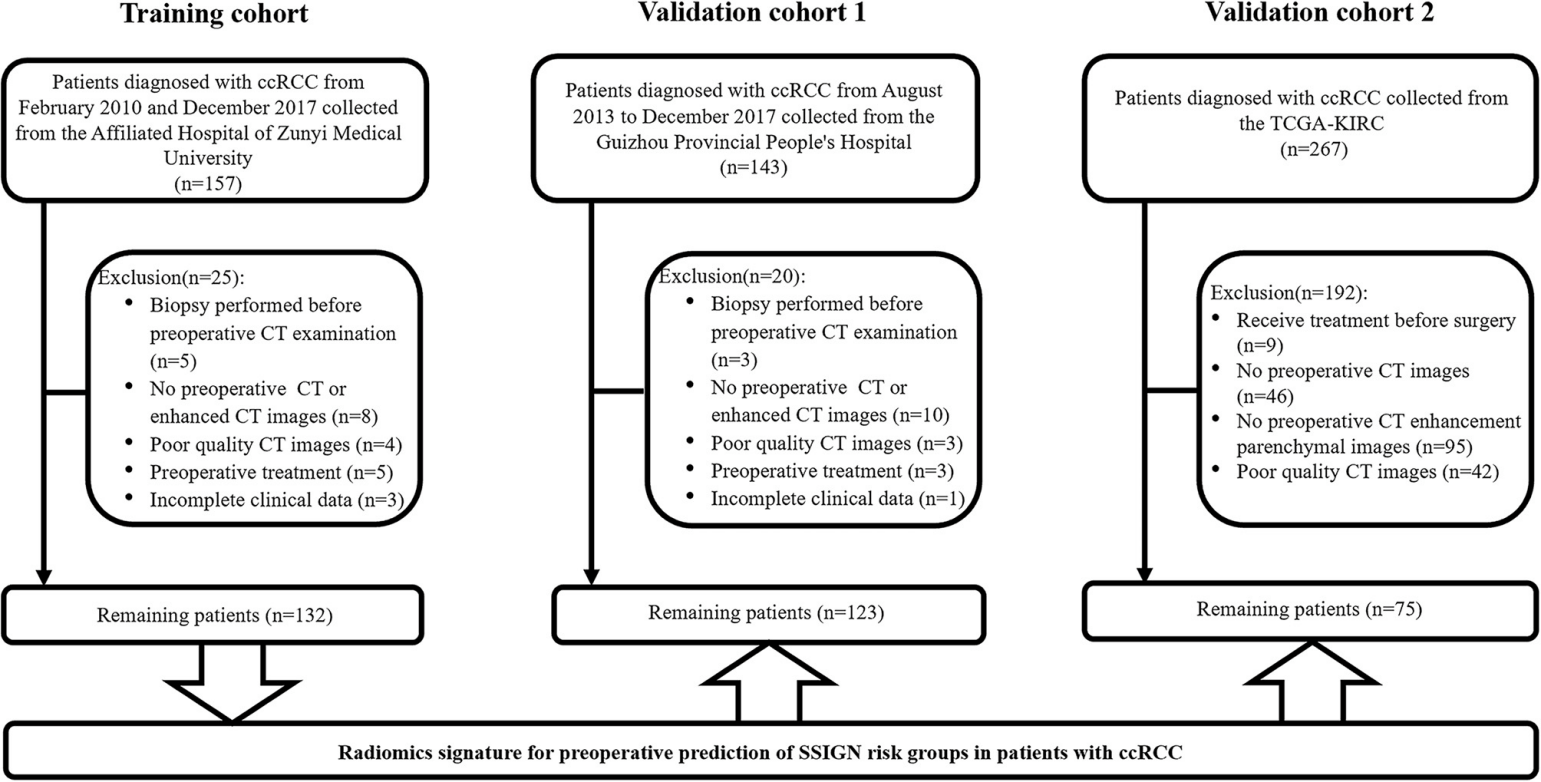
Flowchart of the patient recruitment process.

As shown in Figure 1, a total of 330 eligible patients were enrolled and divided into three independent cohorts as follows: the training cohort consisting of 132 patients (81 low-risk group, 51 intermediate- to high-risk group) from AHZMU; external validation cohort 1 consisting of 123 patients (78 low-risk group, 45 intermediate- to high-risk group) from GZPPH; and external validation cohort 2 consisting of 75 patients (38 low-risk group, 37 intermediate to high-risk group) collected from TCGA-KIRC. There were no significant differences between these cohorts in the SSIGN risk group (*p* > 0.05). The demographics, the clinicopathology characteristics, and the image features of all patients are shown in Table 1.

**Table 1:** Characteristics of ccrcc Patients in the training cohorft, validation cohort 1 and validation cohort 2.

**Characteristics**	**Training cohort (*****n*****=132)**			**Validation cohort 1 (*****n*****=123)**			**Validation cohort 2 (*****n*****=75)**		
	**SSIGN low risk group (*n* = 81)**	**SSIGN intermediate-high risk group (*n* = 51)**	***P*-value**	**SSIGN low risk group (*n* = 78)**	**SSIGN intermediate-high risk group (*n* = 45)**	***P*-value**	**SSIGN low risk group (*n*=38)**	**SSIGN intermediate-high risk group (*n* = 37)**	***P*-value**
Age	56.99 ± 11.39	56.02 ± 16.03	0.708	56.08 ± 13.53	55.93 ± 11.53	0.952	57.95 ± 14.95	60.86 ± 11.55	0.348
Gender			0.714			0.703			0.489
Female	30 (37.04%)	21 (41.18%)		31 (39.74%)	16 (35.56%)		15 (39.47%)	18 (48.65%)	
Male	51 (62.96%)	30 (58.82%)		47 (60.26%)	29 (64.44%)		23 (60.53%)	19 (51.35%)	
tumor	4.25 ± 1.65	6.53 ± 2.20	<0.001^*^	4.15 ± 1.60	6.94 ± 2.18	<0.001^*^	3.97 ± 1.42	7.90 ± 2.54	<0.001^*^
Tumor boundary (%)			0.012^*^			<0.001^*^			<0.001^*^
Circumscribed	74 (91.36%)	38 (74.51%)		72 (92.31%)	27 (60.00%)		38 (100.00%)	23 (62.16%)	
Infiltrative	7 (8.64%)	13 (25.49%)		6 (7.69%)	18 (40.00%)		0 (0.00%)	14 (37.84%)	
Necrosis imaging (%)			0.857			0.010^*^			0.002^*^
Absent	32 (39.51%)	21 (41.18%)		17 (21.79%)	2 (4.44%)		23 (60.53%)	9 (24.32%)	
Present	49 (60.49%)	30 (58.82%)		61 (78.21%)	43 (95.56%)		15 (39.47%)	28 (75.68%)	
Renal vein invasion (%)			0.030^*^			<0.001^*^			<0.001^*^
Absent	75 (92.59%)	40 (78.43%)		77 (98.72%)	34 (75.56%)		38 (100.00%)	28 (75.68%)	
Present	6 (7.41%)	11 (21.57%)		1 (1.28%)	11 (24.44%)		0 (0.00%)	9 (24.32%)	
Collecting system invasion (%)			<0.001^*^			<0.001^*^			0.007^*^
Absent	73 (90.12%)	30 (58.82%)		74 (94.87%)	22 (48.89%)		36 (97.30%)	27 (72.97%)	
Present	8 (9.88%)	21 (41.18%)		4 (5.13%)	23 (51.11%)		1 (2.70%)	10 (27.03%)	
Intratumoral vessels (%)			<0.001^*^			<0.001^*^			0.005^*^
Absent	39 (48.15%)	5 (9.80%)		24 (30.77%)	2 (4.44%)		28 (73.68%)	15 (40.54%)	
Present	42 (51.85%)	46 (90.20%)		54 (69.23%)	43 (95.56%)		10 (26.32%)	22 (59.46%)	
lymphatic metastasis (%)			<0.001^*^			<0.001^*^			0.054^*^
Absent	80 (98.77%)	42 (82.35%)		75 (96.15%)	31 (68.89%)		38 (100.00%)	33 (89.19%)	
Present	1 (1.23%)	9 (17.65%)		3 (3.85%)	14 (31.11%)		0 (0.00%)	4 (10.81%)	
Visual relative enhancement (%)			0.073			0.343			0.931
Hyperattenuating	7 (8.64%)	7 (13.73%)		11 (14.10%)	11 (24.44%)		12 (31.58%)	13 (35.14%)	
Isoattenuating	60 (74.07%)	28 (54.90%)		47 (60.26%)	23 (51.11%)		20 (52.63%)	19 (51.35%)	
Hypoattenuating	14 (17.28%)	16 (31.37%)		20 (25.64%)	11 (24.44%)		6 (15.79%)	5 (13.51%)	
Enhancement pattern (%)			0.362			0.009^*^			0.043^*^
Homogeneous enhancement	31 (38.27%)	14 (27.45%)		28 (35.90%)	10 (22.22%)		18 (47.37%)	8 (21.62%)	
Relatively homogeneous enhancement	25 (30.86%)	16 (31.37%)		34 (43.59%)	14 (31.11%)		8 (21.05%)	8 (21.62%)	
Heterogeneous enhancement	25 (30.86%)	21 (41.18%)		16 (20.51%)	21 (46.67%)		12 (31.58%)	21 (56.76%)	
Tumor Size	3.37 ± 0.96	6.40 ± 2.15	<0.001^*^	3.63 ± 1.17	7.64 ± 2.24	<0.001^*^	3.57 ± 1.15	8.85 ± 3.48	<0.001^*^
WHO/ISUP grading (%)			<0.001^*^			<0.001^*^			0.006^*^
I	20 (24.69%)	3 (5.88%)		15 (19.23%)	1 (2.22%)		10 (26.32%)	5 (13.51%)	
II	53 (65.43%)	24 (47.06%)		60 (76.92%)	19 (42.22%)		16 (42.11%)	14 (37.84%)	
III	8 (9.88%)	19 (37.25%)		3 (3.85%)	19 (42.22%)		12 (31.58%)	8 (21.62%)	
IV	0 (0.00%)	5 (9.80%)		0 (0.00%)	6 (13.33%)		0 (0.00%)	10 (27.03%)	
Coagulative Necrosis.			<0.001^*^			<0.001^*^			0.005^*^
present	10 (12.35%)	41 (80.39%)		5 (6.41%)	32 (71.11%)		11 (28.95%)	23 (62.16%)	
absent	71 (87.65%)	10 (19.61%)		73 (93.59%)	13 (28.89%)		27 (71.05%)	14 (37.84%)	
T stage (%)			<0.001^*^			<0.001^*^			<0.001^*^
T1	76 (93.83%)	23 (45.10%)		77 (98.72%)	11 (24.44%)		36 (94.74%)	6 (16.22%)	
T2	3 (3.70%)	21 (41.18%)		0 (0.00%)	24 (53.33%)		0 (0.00%)	7 (18.92%)	
T3	2 (2.47%)	7 (13.73%)		1 (1.28%)	9 (20.00%)		2 (5.26%)	22 (59.46%)	
T4	0 (0.00%)	0 (0.00%)		0 (0.00%)	1 (2.22%)		0 (0.00%)	2 (5.41%)	
N stage (%)			0.073			0.366			0.002^*^
N1	1 (1.23%)	4 (7.84%)		0 (0.00%)	1 (2.22%)		0 (0.00%)	8 (21.62%)	
N0+Nx	80 (98.77%)	47 (92.16%)		78 (100.00%)	44 (97.78%)		38 (100.00%)	29 (78.38%)	
M stage (%)			<0.001^*^			0.002^*^			<0.001^*^
M0	81 (100.00%)	40 (78.43%)		78 (100.00%)	39 (86.67%)		38 (100.00%)	28 (75.68%)	
M1	0 (0.00%)	11 (21.57%)		0 (0.00%)	6 (13.33%)		0 (0.00%)	9 (24.32%)	
TNM stage (%)			<0.001^*^			<0.001^*^			<0.001^*^
I	75 (92.59%)	14 (27.45%)		77 (98.72%)	11 (24.44%)		36 (94.74%)	4 (10.81%)	
II	3 (3.70%)	19 (37.25%)		0 (0.00%)	21 (46.67%)		0 (0.00%)	5 (13.51%)	
III	3 (3.70%)	7 (13.73%)		1 (1.28%)	7 (15.56%)		2 (5.26%)	19 (51.35%)	
IV	0 (0.00%)	11 (21.57%)		0 (0.00%)	6 (13.33%)		0 (0.00%)	9 (24.32%)	

* *P < 0.05 means statistical significance*.

*Data are in n (%) unless otherwise indicated*.

*Categorical variables are compared using chi-square tests or Fisher exact tests, while continuous variables are compared using t-test or Mann-Whitney U-test, as appropriate*.

### Radiomic Signature Construction

A total of 1,218 radiomic features were extracted from the nephrographic phase contrast-enhanced CT images with 1144 radiomics features remaining by eliminating the radiomic features with non-robustness (ICC < 0.75) between the inter- and intra-observers. Then, 16 SSIGN risk group-related radiomic features with non-zero coefficients were screened using the LASSO regression analysis. A radiomic signature based on the above radiomic features was constructed via the LASSO logistic regression model in the training cohort. The Rad score calculation formula is shown in Supplementary S2, and the optimal risk cutoff value of the Rad score was 0.352 according to the maximized Youden index in the training cohort. Consequently, a statistically significant difference was observed in the Rad scores [median (interquartile range)] between the low-risk group and intermediate- to high-risk group in the training cohort [0.097 (0–0.346) vs. 0.744 (0.353–1), respectively, *p* < 0.001]. This difference was confirmed in external validation cohort 1 [0.095 (0.001–0.3441) vs. 0.727 (0.727–0.378), respectively, *p* < 0.001] and in external validation cohort 2 [0.086 (0–0.311) vs. 0.813 (0.372–1), respectively, *p* < 0.001]. Finally, the radiomic signature demonstrated a favorable predictive performance with an AUC of 0.940 [95% confidence interval (CI), 0.884–0.973] in the training cohort, 0.876 (95% CI, 0.811–0.942) in external validation cohort 1 and 0.928 (95% CI, 0.844–0.975) in external validation cohort 2.

### Image Feature Model and Fusion Model Construction

In the univariate analysis, the image features of the tumor boundary, the renal vein invasion, the collecting system invasion, the intra-tumoral vessels, and the enhancement pattern were significantly different between the SSIGN low-risk group and intermediate- to high-risk group (*p* < 0.05). There was only one image feature, intra-tumoral vessels (OR 11.463 [9.702–13.226], *P* < 0.001), as an independent predicted factor for the SSIGN intermediate- to high-risk groups by applying multivariate logistic regression analysis. Consequently, an image feature model was developed based on the intra-tumoral vessels and yielded an AUC of 0.708 (95% CI, 0.625–0.787) in the training cohort, 0.630 (95% CI, 0.538–0.715) in external validation cohort 1 and 0.666 (95% CI, 0.547–0.771) in external validation cohort 2.

In addition, a fusion prediction model was constructed combining the radiomic signature and the independent predictor which demonstrated AUCs of 0.942 (95% CI, 0.887 to 0.975), 0.876 (95% CI, 0.808–0.945) and 0.920 (95% CI, 0.834–0.970), respectively, for the training and external validation cohorts.

### Model Evaluation and Model Comparison

**Figure 2:**
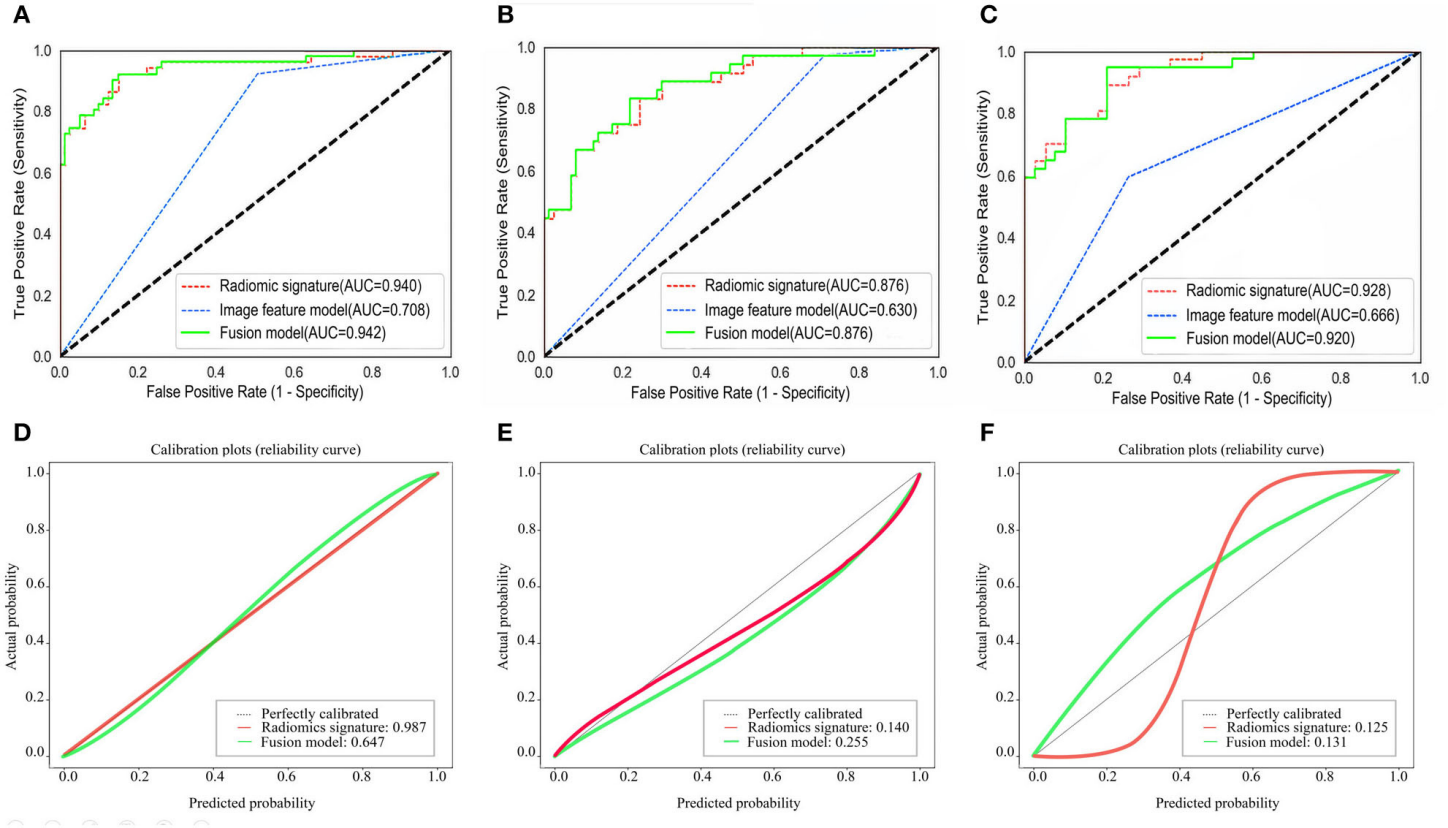
Comparison of ROC curves between radiomic signature, image feature model, and fusion model for prediction of tumor necrosis in the training cohort **(A)**, the validation cohort 1 **(B)**, and the validation cohort 2 **(C)**. The three colors of the curves represent different models: green, radiomics signature; blue, image feature model; red, fusion model. Calibration curves of the radiomic signature, fusion model in the training cohort **(D)**, the validation cohort 1 **(E)**, and the validation cohort 2 **(F)**, respectively. Calibration curves show the calibration of the nomogram in terms of agreement between the predicted probability of SSIGN risk group and actual probability. The 45 black lines represent a perfect prediction, and the green and red lines represent the predictive performance of the radiomic signature and the fusion model, respectively. The closer the dotted line fit is to the ideal line, the better the predictive accuracy of the model is.

The ROC curves of the radiomic signature, the image features, and the fusion model are demonstrated in Figures 2A–C and the predicted performance summarized in Table 2 for all cohorts. Through the DeLong test, the results showed that the AUCs of the radiomic signature and the fusion model exceeded that of the image feature model (*p* < 0.001 and *p* < 0.001, respectively, in all cohorts), while no significant differences in the AUC values were discovered between the radiomic signature and the fusion model in the training and external validation cohorts (*p* = 0.575, 1.000, 0.304), summarized in Table 3. The results indicated that they were equally effective in the discrimination performance between the SSIGN low-risk and intermediate- to high-risk groups.

**Table 2:** Predictive performance of the radiomics signature, image feature model, fusion model in all cohorts.

**Model**	**Trainning cohort (***n*** = 132)**					**Validation cohort 1 (***n*** = 123)**					**Validation cohort 2 (***n*** = 75)**				
	**AUC**	**95%CI (AUC)**	**acurracy**	**sensitivity**	**spencificity**	**AUC**	**95%CI (AUC)**	**acurracy**	**sensitivity**	**spencificity**	**AUC**	**95% CI(AUC)**	**acurracy**	**sensitivity**	**spencificity**
Radiomics signature	0.940	0.884–0.973	87.88%	85.19%	92.16%	0.876	0.811–0.942	78.86%	81.61%	72.22%	0.928	0.844–0.975	81.33%	94.74%	67.57%
Image feature model	0.708	0.625–0.787	65.91%	49.38%	92.16%	0.630	0.538–0.715	48.78%	28.74%	97.22%	0.666	0.547–0.771	66.67%	73.68%	59.46%
Fusion model	0.942	0.887–0.975	87.88%	85.19%	92.16%	0.876	0.808–0.945	80.49%	82.76%	75.00%	0.920	0.834–0.970	80.00%	94.74%	64.86%

**Table 3:** Model prediction performance comparison.

	**Training cohort**	**Validation cohort 1**	**Validation cohort 2**
Radiomics signature vs. image feature model	*p* < 0.0001	*p* < 0.0001	*p* < 0.0001
Fusion model vs. image feature model	*p* < 0.0001	*p* < 0.0001	*p* < 0.0001
Radiomics signature vs. fusion model	*p* = 0.575	*p* = 1.000	*p* = 0.304

The calibration curves in all the cohorts are illustrated in Figures 2D–F. The calibration curve and the Hosmer–Lemeshow test revealed that the radiomic signature and the fusion model both demonstrated an excellent agreement between the expected and predicted consistency probabilities in training cohorts (*p* = 0.987 and *p* = 0.647). The favorable calibration was further verified in external validation 1 cohort (*p* = 0.140 and *p* = 0.255) and external validation 2 cohort (*p* = 0.125 and *p* = 0.131).

**Figure 3:**
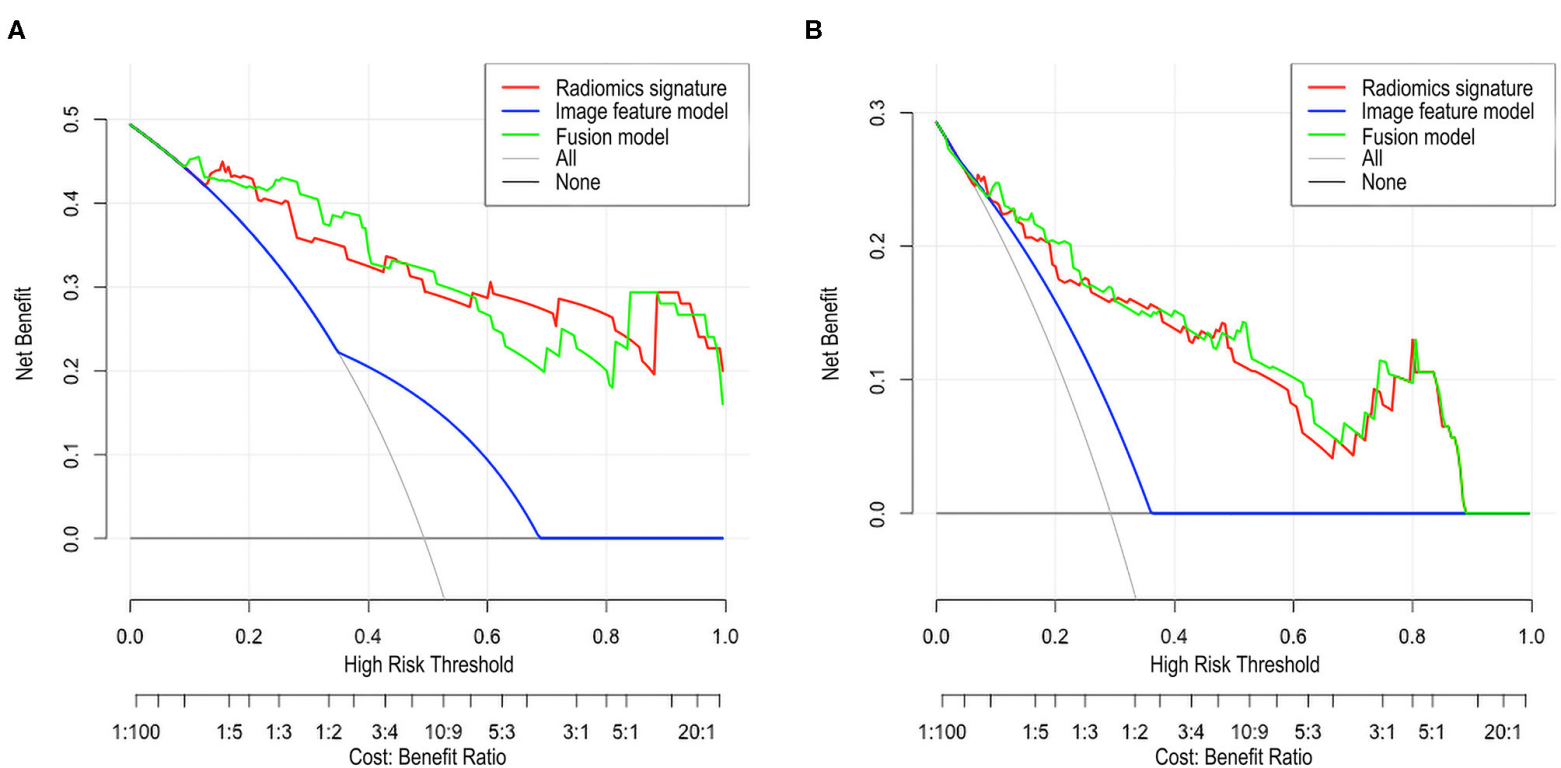
Decision curve analysis (DCA) for each model in the validation cohorts. The DCA demonstrated that if the threshold probability was >12% in the validation cohort 1 **(A)** and in the validation cohort 2 **(B)**, the application of radiomics signature and fusion model to predict SSIGN risk group performance equals and adds more benefit than does the image model and treats all or none of the patients.

The DCA of the radiomic signature, the image features, and the fusion model are presented in Figure 3. The radiomic signature and the fusion model provided more net benefits than the image model and the treat-all or treat-none scheme, and the two models showed no significant differences in the threshold probability >12% in the external validation cohorts, thus indicating that both models attained similar performance with regard to their clinical application.

## Discussion

In this multicenter study, a radiomic signature was proposed with an excellent predictive accuracy to discriminate SSIGN low-risk and intermediate to high-risk groups in patients with ccRCC. This significantly outperformed the image feature model and showed similar performance with the fusion model in terms of the discrimination, calibration, and clinical value in the training cohort and both validation cohorts. The results demonstrated the feasibility and reproducibility of the radiomic signature in preoperative SSIGN risk assessment between different centers for ccRCC patients.

A radiomic signature in the current study was constructed using eleven selected features including shape features, first-order feature, and texture features. Pathologically, tumor size is an important indicator of tumor staging and associated with higher nuclear grade, more histologic necrosis, and sarcomatoid changes (15, 20–22). As a consequence, the shape features, especially the major axis length, which is the largest axis length of the tumor, contributed to predicting the SSIGN risk groups. The only first-order feature was kurtosis, a statistical parameter of peakedness or the sharpness of the histogram, which increased with lower heterogeneity (23). In agreement with this principle, the ccRCC with low risk demonstrated higher kurtosis values when compared to high-risk ccRCC, suggesting a more homogeneous pattern within the pixels in the SSIGN low-risk group. Compared with the above two types of features, the texture features yielded a better diagnostic performance according to the LASSO coefficients. The texture features were used to describe the patterns or spatial distributions of voxel intensity and proved to be an efficient approach in characterizing tumor macroscopic heterogeneity, which is a potential representation of tumor aggressiveness (24, 25). In previous studies, the differentiated distribution of texture features can be detected between low and high WHO/ISUP grade ccRCC, sarcomatoid, and non-sarcomatoid RCC (12, 15). Therefore, the texture features provide important supplementary information for other features and constitute the most relevant feature set for SSIGN risk prediction.

Consistent with the previous study, the image feature model constructed and based on the intra-tumoral vessels was significantly worse than the radiomic signature in discriminating performance, further proving that the radiomic features can produce more detailed phenotypic information about a tumor hard to detect with the naked eye (10, 26, 27). Furthermore, a fusion model was constructed by integrating the radiomics signature and the image feature. However, there was no significant difference between the radiomic signature and the fusion model in the discrimination, calibration, and clinical value on account of which the image feature, the intra-tumoral vessels, could not add any incremental value to the radiomic signature. Therefore, this study considered the single radiomic signature with improved efficiency, reproducibility, and consistency and pipeline systems to potentially provide an easy-to-use tool to predict the SSIGN risk groups for patients with ccRCC.

In different centers, there was a great challenge in validating the radiomic models reflecting the tumor's invasiveness by predicting a single pathological index. This was because the evaluation of the pathological indicator may be differed among different pathologists (28, 29). Unlike these, the SSIGN score in this study had a better credibility and generalization among the different centers as the multi-indicator comprehensive model could have reduced the influence of the errors and bias caused by a single indicator used for the diagnosis. Additionally, in order to ensure the generalizability and reproducibility of the radiomic signature, this study was constructed using a large sample size and validated by two independent external datasets, including those of the TCGA-KIRC. Therefore, the radiomic signature capable of predicting the SSIGN risk group has great clinical and practical value.

Overall, our study has important practical implications because SSIGN is one of the commonest used prediction systems for the overall survival prognosis of ccRCC patients. However, percutaneous biopsy serves as a standard method for tumor aggressiveness assessment *in vivo*. However, this kind of biopsy cannot deliver a SSIGN score and is limited by sampling bias, unsatisfactory accuracy, and the use of an invasive method (30). Considering the favorable performance in predicting the SSIGN risk groups in the multicenter datasets, radiomic analysis may be an alternative method for the assessment of the aggressiveness of ccRCCs and could play a more key role in the choice of optimal treatment methods for ccRCC patients before surgery. In addition, radiomic analysis with its non-invasive nature and automated analysis can be seen as a promising tool to repeatedly assess patients with ccRCC being treated conservatively, such as them being under active surveillance and using ablative therapies during follow-up.

There were several limitations in this study. First, although these models were satisfactory when it came to accuracy in the two independent external validation cohorts, the robustness and repeatability should be validated by a larger prospective cohort. Second, this study only focused on the value of radiomics in the discrimination of SSIGN low-risk and intermediate- to high-risk groups due to the limited sample size and the unbalanced patient distribution. However, the prediction of more at-risk subgroups based on the SSIGN score may be of greater value in the diagnosis and treatment of ccRCC patients. Third, there were greater heterogeneities in the CT scan equipment and the parameters between inter-central and intra-central, especially in the TCGA-KIRC cohort. Fourth, the loss of the interpretability and explainability of the radiomic features remained as an important challenge for the application of the radiomic signature clinically.

In conclusion, this current study proposed a CT-based radiomic signature that demonstrated satisfactory predictive performance in distinguishing SSIGN low-risk group and an intermediate- to high-risk group of ccRCC preoperatively. As a quantitative and non-invasive predictive tool, a radiomic signature is expected to further facilitate clinical decision-making.

## Data Availability Statement

Publicly available datasets were analyzed in this study. This data can be found here: https://wiki.cancerimagingarchive.net/.

## Ethics Statement

Permission for the study was granted by the ethics committee of GZPPH and the requirement for patient informed consent was waived because it was a retrospective study.

## Author Contributions

YJ: data processing and thesis writing. WL and QC: data collation and analysis. CH: data processing. CT, XZ, and HL: image analysis and lesion segmentation. YCao, YChen, YY, and YB: pathological review and analysis. CL: case screening. YL: statistical analysis. TZ: experimental design. RW: experimental design and review. All authors contributed to the article and approved the submitted version.

## Conflict of Interest

CH and YL were employed by the Beijing Deepwise & League of PHD Technology Co. Ltd,. The remaining authors declare that the research was conducted in the absence of any commercial or financial relationships that could be construed as a potential conflict of interest.

## Abbreviations

RCC: Renal cell carcinoma
ccRCC: Clear cell renal cell carcinoma
SSIGN: Stage size, grade, and necrosis
CT: Computed tomography
GZPPH: Guizhou Provincial People's Hospital
AHZMU: Affiliated Hospital of Zunyi Medical University
TCGA: The Cancer Genome Atlas
TCGA-KIRC: The Cancer Genome Atlas-Kidney Renal Clear Cell Carcinomas
WHO: World Health Organization
ISUP: International Society of Urological Pathology
IBSI: Image Biomarker Standardization Initiative
ROI: Region of interest
ICC: Intra-class correlation coefficient
Rad score: Radiomic score
ROC: Receiver operating characteristics
AUC: Area under the curve
DCA: Decision curve analysis
LASSO: Least absolute shrinkage and selection operator.
